# Natalizumab in Multiple Sclerosis: Long-Term Management

**DOI:** 10.3390/ijms18050940

**Published:** 2017-04-29

**Authors:** Marinella Clerico, Carlo Alberto Artusi, Alessandra Di Liberto, Simona Rolla, Valentina Bardina, Pierangelo Barbero, Stefania Federica De Mercanti, Luca Durelli

**Affiliations:** Clinical and Biological Sciences Department, University of Torino, Orbassano (TO) 10043, Italy; marinella.clerico@unito.it (M.C.); caartusi@gmail.com (C.A.A.); ale.dili@hotmail.it (A.D.L.); simona.rolla@unito.it (S.R.); valentina.bardina87@gmail.com (V.B.); p.barbero@isiline.it (P.B.); luca.durelli@unito.it (L.D.)

**Keywords:** natalizumab, natalizumab discontinuation, long term safety, natalizumab management, progressive multifocal leukoencephalopathy, therapeutic switch

## Abstract

Natalizumab is a monoclonal antibody highly effective in the treatment of relapsing remitting multiple sclerosis (RRMS) patients. Despite its effectiveness, there are growing concerns regarding the risk of progressive multifocal leukoencephalopathy (PML), a brain infection caused by John Cunningham virus (JCV), particularly after 24 doses and in patients who previously received immunosuppressive drugs. Long-term natalizumab treated, immunosuppressive-pretreated, and JCV antibody-positive patients are asked to rediscuss natalizumab continuation or withdrawal after 24 doses. Until now, there has not been a clear strategy that should be followed to avoid PML risk and in parallel reduce clinical and radiological rebound activity. In this review, we analyzed the results of clinical trials and case reports in relation to the following situations: natalizumab continuation, natalizumab discontinuation followed by full therapeutic suspension or switch to other first or second line MS treatments. Quitting all MS treatment after natalizumab increases MS activity occurrence. The results regarding the therapeutic switch are not homogeneous, so at the moment there are no established guidelines regarding natalizumab treatment after 24 administrations; the choice is currently based on the professional experience of the neurologist, and on patients’ clinical features and preferences.

## 1. Introduction

Multiple sclerosis (MS) is a chronic inflammatory demyelinating disease of the Central Nervous System (CNS) [1]; it is a frequent illness, affecting more than 2.3 million people in the world [2]. It is estimated that 85–90% of MS patients have a relapsing remitting (RR) course [2], characterized by the recurrent appearance of inflammatory lesions in the brain and in the spinal cord, causing demyelination plaques and, finally, potential axonal loss [3]. A crucial and early role in the demyelinating process is the lymphocytes migration across the blood brain barrier (BBB) [4]. Several studies [5,6,7,8] demonstrated that one of the mechanisms involved in the adhesion and migration of lymphocytes to the CNS is related to the interaction of the α4β1 integrin expressed on their surface with the vascular-cell adhesion molecule 1 (VCAM-1) on the surface of vascular endothelial cells of CNS blood vessels. It has been postulated that α4β1 integrin may interact with other proteins, such as fibronectin [8] and osteopontin [9], thus promoting the survival and activation of the leukocytes inside the CNS [10].

Natalizumab (Tysabri^®^; Biogen-Idec, Cambridge, MA, USA) is a humanized anti–α4 integrin monoclonal antibody; it binds the α4 subunit of α4β1 and α4β7 integrins, blocking the binding to their endothelial receptors, thereby attenuating the CNS inflammation [11]. In addition, natalizumab acts also by inhibiting the interaction between α4-positive leukocytes, fibronectin, and osteopontin [12]. Natalizumab was initially approved by the US Food and Drug Adminsitration (FDA) in 2004 for RRMS, upon the interim results of two phase-III trials, the AFFIRM and SENTINEL [12,13], but in 2005 it was taken off the market after the occurrence of three cases of progressive multifocal leukoencephalopathy (PML): two in MS patients and one in a Crohn’s disease (CD) patient [14,15,16]. 

After completing data analysis of the phase III trials [12,13] and in consideration of natalizumab great efficacy, it was released on the market in the European Union in 2006 in association to a Global Risk Management Plan. In particular, with the help of the FDA, the “TOUCH^®^” program (TYSABRI Outreach: Unified Commitment to Health Prescribing Program) [17] has been developed in order to facilitate its appropriate use. In particular, it is a restricted distribution program focused on safety; according to this plan, only prescribers and patients enrolled in the TOUCH prescribing program could prescribe and receive natalizumab and only certain pharmacies and infusion sites are approved to dispense and infuse it [17]. As of September 2016, approximately 161,300 patients received natalizumab in post-marketing setting worldwide, and as of December 2016, 698 treated patients reported a confirmed PML diagnosis [18]. PML is an infective and demyelinating CNS disease, due to the polyomavirus John Cunningham (JCV) reactivation [19,20]. Primary infection with JCV typically occurs in early life and it is frequently asymptomatic; then JCV presumably remains latent in various tissues, such as the kidneys, bone marrow, and lymphoid tissue [21,22]. Most of the studies observed a prevalence of around 60–70% of detectable antibodies against JCV in the general population, with seroprevalence increasing with the age [21]. PML probably occurs as the interaction between JCV seropositivity and patient’s features [22]: i.e., the disease typically affects heavily immunosuppressed patients [22].

No PML cases have been reported in MS patients before the introduction of natalizumab [23]; in case of JCV reactivation, the infection affects the myelin-producing oligodendrocytes, resulting in severe demyelination [24,25,26,27]. After the analysis of PML cases, it seems that the infection is more widespread in the brain [28,29,30,31]. PML can be supposed on the basis of the clinical presentation and by the neuroimaging findings and should be confirmed by the detection of the virus in the CSF through the Polymerase Chain Reaction (PCR) test; the gold standard for the diagnosis of PML is the detection of JCV in histopathology analysis of biopsy material [32,33]. At the moment, there are no proven specific and effective treatment for PML; the clinical outcome depends entirely on the personal immune reconstitution to respond to JCV [34]. The main and mandatory intervention in case of suspected PML is the immediate withdrawal of natalizumab and, after PML diagnosis confirmation, some studies suggest to accelerate the removal of the residual plasmatic quote of natalizumab using the plasma exchange therapy (PLEX) [35,36].

In natalizumab MS treated patients, known risk factors for JCV reactivation are concomitant or previous immunosuppression and natalizumab exposure duration, particularly after the 24th administration [22]. More recently, the anti-JC virus antibody level in serum or plasma has been identified as a further risk of natalizumab-associated PML [37]. According to these data, European Medicines Agency (EMA) has recently updated the estimate risk for PML in seropositive JCV antibody patients treated with natalizumab [38]; the risk is small at antibody index values of 0.9 or less (0.1–0.6/1000), and increases substantially in patients with index values above 1.5 who have been treated with natalizuab for more than 24 administrations (0.9–10/1000) [38]. Data from Plavina and colleagues [37] suggest that anti-JCV antibody index may fluctuate over time; during a period of 18 months, they observed that the 87% of patients with anti-JCV antibodies negative test at baseline remained negative during subsequent testing; the 97% of patients who tested anti-JCV antibody-negative at baseline remained below an anti-JCV antibody index threshold of 1.5, even after conversion; the 3% of patients seroconverted to an index >1.5 [37]. The 18% of patients who had an index >1.5 maintained this level for at least six months prior to a diagnosis of PML and during subsequent tests [37].

Patients with previous immunosuppressant therapy after 24 doses have a PML risk ranging from 0.4/1000 to 10/1000 [38]. Following EMA recommendations, after the 24th natalizumab dose, patients should be informed again about the risk of PML related to natalizumab and they are asked to provide a standardized written consent form to continue this therapy [39]. Moreover, patients should be informed to be vigilant about the risk of PML for up to six months after discontinuation of natalizumab [39]. At the 24th natalizumab dose, patients should evaluate again with the neurologist the opportunity to continue natalizumab, to switch to any other first or second line MS treatment or, alternatively to quit all therapies. Several studies showed that after natalizumab discontinuation disease activity got worse than pre-natalizumab status [40,41,42], indicating a rebound effect, similar to an immune reconstitution inflammatory syndrome (IRIS) [43].

Besides the risk of PML, other circumstances should lead to the decision to stop natalizumab therapy, such as the detection of anti-natalizumab antibodies, incomplete efficacy, tolerability matters, or patient preference for oral therapies [44].

## 2. Literature Review Section

For this review, a PubMed search was performed using the terms “natalizumab”, “natalizumab AND multiple sclerosis”, “natalizumab management”, “natalizumab AND withdrawal”, “natalizumab AND discontinuation”, “natalizumab AND safety”, “natalizumab AND PML”, “natalizumab AND safety” without time restriction.

### 2.1. “Drug Holiday” after Natalizumab Withdrawal

Immunological data from Stüve et al. [45] argue that natalizumab should maintain sustained efficacy even after discontinuation. However, it is known that the suspension of natalizumab may expose to a higher risk of clinical and radiological disease activity. O’Connor et al. [46] retrospectively analyzed a large cohort of RRMS patients from the AFFIRM [12], SENTINEL [13], and GLANCE [47] trials, who suspended voluntarily natalizumab dosing. In this meta-analysis a total of 1866 patients were enrolled and observed for eight months; 544 patients, who did not receive an alternative drug after natalizumab discontinuation, experience a MS activity recurrence with a peak after 4–7 months. In the RESTORE study [48] (a randomized, partially placebo-controlled study to evaluate the effect on MS disease activity of a 24-week interruption in natalizumab treatment), 7 out of 41 patients experienced MS activity during the "drug holiday” period. A recent multicenter study [49] on 124 RRMS patients who had reached clinical and radiological stability after 24 natalizumab courses demonstrated a one-year four-fold higher Annualized Relapse Rate (ARR) (*p* = 0.002) in 73 natalizumab withdrawal patients compared with 35 natalizumab continuers; also Magnetic Resonance Imaging (MRI) activity was significantly higher in withdrawal patients (*p* = 0.03). No rebound activity, defined as a higher individual relapse rate after cessation of natalizumab than before natalizumab [50], was observed in patients discontinuing natalizumab [49]. Similarly, Lo Re et al. [51] reported data on natalizumab continuation/discontinuation in 132 MS patients, of which 37 patients remained therapy free: therapy free patients had a statistically significant higher risk of both clinical (*p* < 0.001) and radiological (*p* < 0.001) relapses if compared with natalizumab continuers after an one-year observation. TYSEDMUS was an observational, prospective, multicenter, nationwide, longitudinal French study of 4055 MS patients aimed to evaluate the disease activity after natalizumab discontinuation [52]. A total of 198 patients remained therapy free for at least one year after natalizumab discontinuation; their ARR was 0.65, lower than before natalizumab start, not confirming the hypothesis of a rebound effect [52]. Killestein et al. [43] followed-up with 10 MS patients for six months after natalizumab discontinuation; they observed the occurrence of both clinical and radiological activity in 7 out of 10 patients.

Iaffaldano and colleagues [53] recently showed that the positive effects of natalizumab on cognitive functions were lost in a group of 30 patients discontinuing natalizumab for one year; indeed, the cognitive impairment index significantly increased in comparison to the group of 28 patients who continued natalizumab (*p* < 0.0001), and this worsening went along with the clinical and radiological disease reactivation: the mean ARR significantly increased during the first year after natalizumab discontinuation (*p* = 0.04), but it was also significantly lower than the ARR in the year prior natalizumab start (*p* = 0.003) [53]. A recent study [54] analyzed two ways of natalizumab discontinuation, immediate versus tapered down, and measured the disease activity during a 12-month follow-up period. This study showed a higher relapse rate in the group with sudden natalizumab discontinuation, compared to the to the tapering group (*p* = 0.007); most of the relapses occurred within three months of discontinuation (*p* = 0.012). The immediate discontinuation group showed also a significant higher number of new T2 lesions within 6–12 months after discontinuation (*p* = 0.025) [54].

Vellinga and colleagues [55] analyzed 21 patients after natalizumab withdrawal; during the study period the median ARR was lower than in pre-natalizumab condition; however an increased number of active lesions were observed, in comparison with the pre-natalizumab status. 

### 2.2. Switch to Other MS Therapies after Natalizumab Withdrawal

Clerico and colleagues [49] showed that, during a 12 months follow-up period, the mean ARR was about three times higher in 16 natalizumab switchers patients (*p* = 0.05). The new treatment (interferons-IFN, glatiramer acetate-GA) was begun immediately after natalizumab withdrawal, whereas the two patients switching to fingolimod had required a three-month washout period, according to the recommendations [49]. In 2015 Lo Re et al. [51] analyzed the effect of switching from natalizumab to other first or second line therapies: 57 patients switched to fingolimod, 16 to first line therapies (IFN, GA, teriflunomide, azathioprine), 7 to rituximab, 4 to immunosuppressive agents (cyclophosphamide or mitoxantrone), and 2 to autologous hematopoietic stem cell transplant. A higher clinical and radiological activity was observed in the first line therapy groups, but this result did not reach the significant threshold [51]. The 17.5% and 23.6% of patients who switched to fingolimod had respectively a clinical or radiological reactivation [51]. Sangalli and colleagues [42], in a prospective study of 110 patients who stopped natalizumab after at least 12 infusions, demonstrated that 30% of patients who received an alternative disease modifying therapy after natalizumab withdrawal had a significantly higher probability to remain “disease-activity free” compared to treatment-free patients (30% versus 0%). There were no significative differences regarding the type of drug after natalizumab, whether they were immunomodulants as IFN and GA or a second line therapy as fingolimod [42]. A recent study [56] analyzed the risk for relapse in patients switching from natalizumab to fingolimod, IFN or GA. They observed a 64% reduction of the adjusted-risk for relapse in patients switching to fingolimod in comparison to those switching to IFN or GA (*p* < 0.0001) [56]. Similarly, Villaverde-Gonzàlez and colleagues [57] followed for 12 months 21 MS patients treated with natalizumab for at least one year with subsequent switch to IFN or GA. At the end of the study period, they did not found significant differences in the ARR compared to natalizumab treatment period (*p* = 0.083); IFN and GA had positive effects also on MRI outcomes, as 62.5% of patients had no evidence of MRI activity after natalizumab discontinuation [57]. On the other hand, O’Connor and colleagues [46] and the RESTORE study [48] showed an increase of MS disease activity after natalizumab withdrawal regardless the switch to IFN or GA.

A case report regarding the switch to another first line therapy, the dimethyl-fumarate, reported on a 21-year-old woman with highly active RRMS who developed both a clinical and radiological rebound five months after natalizumab discontinuation [58].

Kappos and colleagues [44] performed a double-blind, randomized, parallel group study, with the objective of determining the optimal timing for starting fingolimod after natalizumab withdrawal. The results provides Class II evidence that in patients switching from natalizumab to fingolimod, a shorter natalizumab wash out period (between 8–12 weeks after natalizumab withdrawal) is associated with a lower disease activity [44]. A study [59] analyzed the recurrence of disease activity in 15 RRMS patients after two distinct natalizumab withdrawals: patients started INF or GA after the first withdrawal and a second line therapy (mostly fingolimod) after the second one. The study demonstrated the occurrence of disease activity after each natalizumab discontinuation, regardless of the new therapeutic approach chosen [59]. Similarly, the radiological disease activity also increased after natalizumab withdrawal and median time to disease recurrence was similar after both natalizumab discontinuations (*p* = 0.57) [59]. A multicentre Swedish study [60] on 256 RRMS natalizumab patients switching to fingolimod or rituximab evidenced a significant improved effectiveness and tolerability of rituximab compared with fingolimod. A recent study of Malucchi and colleagues [61] on 16 high-risk PML patients switching from natalizumab to alemtuzumab, after a median wash-out period of 70 days (range 41–99 days), showed that alemtuzumab could control the disease activity in patients who stopped taking natalizumab.

### 2.3. Pulsed Corticosteroid Treatment after Natalizumab Discontinuation

In 2011 Borriello and colleagues [62] performed a prospective post-marketing study on 23 MS patients after natalizumab discontinuation (range: 90–150 days): despite the monthly pulsed steroid therapy, seven patients had a radiological relapse and four of them had a concomitant clinical relapse [62].

More recently, a study [63] analyzed the effect of pulsed corticosteroid treatment on 20 MS patients during a six-month washout period after natalizumab discontinuation. Patients received monthly intravenous methylprednisolone (1000 mg/infusion) and received regular clinical and radiological assessment. During the six-month washout period, only one patient out of 10 had a mild sensory relapse associated with MRI activity in the group treated with methylprednisolone [63]. In the control group of untreated 10 patients, one developed several active lesions in brain MRI and another one had a severe relapse. These data suggest that monthly methylprednisolone treatment in the washout period could determine a clinically stable disease phase (*p* < 0.01), allowing a more safe transition to another therapy [63]. Rossi and colleagues [64] highlighted a possible paradox effect of corticosteroids associated to GA after natalizumab discontinuation, both on clinical and MRI outcomes.

### 2.4. Natalizumab Management during Pregnancy

MS is at least two to three times more common in women than in men [2], thereby long term MS management could involve pregnancy. To date, natalizumab is a pregnancy category “C” drug [65], however, no clear teratogen effects have been observed nowadays, and normal outcomes of pregnancy have been reported in some published cases of patients treated for the gestational period [66,67]. By contrast, a study with a small case series found mild hematological alterations in 10 out of 13 children of mothers receiving natalizumab during the third pregnancy trimester [68]. Ebrahimi and colleagues [69] performed a study on 101 German MS patients exposed to natalizumab during the first trimester of pregnancy and compared to MS-matched and healthy control groups. Results showed, compared to the healthy group, a significant higher miscarriage rate and lower birth weights among both MS groups, but there was not a significant difference between the natalizumab and the MS-matched groups. More recently, a study [70] was undertaken to evaluate pregnancy outcomes on 349 MS and 6 CD patients treated with natalizumab at any time within three months prior to conception or during pregnancy. A global, observational exposure registration pregnancy registry was created: the Tysabri Pregnancy Exposure Registry [70]. Birth defects were analysed and coded according to the Metropolitan Atlanta Congenital Defects Program (MACDP) classification of birth defects. Although the overall rate of birth defects were higher than that observed by the MACDP, the data from Friend and colleagues [70] did not show a specific pattern of malformations possibly related to a drug effect; the spontaneous abortion rate was comparable with that of the general population.

### 2.5. Immunological Consequences of Natalizumab Withdrawal

Larochelle and colleagues [71] report recently a case of fatal CNS inflammatory demyelination after natalizumab discontinuation; the post-mortem pathological analysis of the brain detected numerous active inflammatory demyelinating lesions characterized by the immunopathological pattern II. The Authors found many monocytes, macrophages, and B cells in CNS parenchyma compared to the CSF [71]. The lesions were enriched with numerous plasma cells; CD8 T lymphocytes were prevalent in the parenchyma, conversely the CD4 T lymphocytes were predominant in the CSF [71]; these lymphocytes expressed high levels of pathogenic molecules such as granzyme B, IFN-γ, and interleukin (IL)17 [71]. To determine the role of T helper 17 (Th17) cells in MS reactivation after natalizumab discontinuation, Haas et al. [72] analyzed Th17 cells and IL-17 levels in the peripheral blood of 57 MS patients, before, during, and after natalizumab treatment. The results showed that Th17 and IL-17 levels increased during prolonged natalizumab treatment, returned to baseline levels after withdrawal and resulted almost undetectable in patients who experienced relapses during the natalizumab washout period [72]. Looking for a biomarker for a safe and effective change from natalizumab to fingolimod, Harrer and colleagues [73] monitored five parameters related to pharmacokinetic and pharmacodynamic effects of the two therapies; they studied 12 MS patients previously treated with natalizumab (for at least 12 months) during the eight-week wash out period and the six months of fingolimod treatment. The authors found an high interindividual variability and the only linkage with MS reactivation was an higher frequencies of memory CD8 lymphocytes after six months on fingolimod, thus, none of the analyzed parameters showed a potential role as a prognostic biomarker for the outcome of the switch [73].

## 3. Discussion

Natalizumab is a highly effective treatment for RRMS. As shown by two big open label, post-marketing studies, the STRATA [74] and the TOP [75] studies, natalizumab remains highly effective over time. In the STRATA study [74] the ARR throughout the 240-week study period, the relapse rate during the first 12 months and the Expanded Disability Status Scale (EDSS) score at every time point up to week 240 were significantly lower in patients treated with natalizumab than placebo. The TOP study [75], that evaluated the effect of natalizumab on disability progression beyond two years of treatment in clinical practice, showed that patients who remained on continuous natalizumab therapy for more than four years had sustained and potentially enhanced reductions in EDSS worsening over time.

Over the last few years the issue of the continuation of treatment with natalizumab after 24 doses is strongly emerging. Several studies have been developed to analyze the best strategy to reduce the risk of PML and, at the same time, to minimize the clinical and radiological relapses correlated to natalizumab, including the risk of rebound. However, in some conditions natalizumab discontinuation is highly recommended, especially in MS patients with high JCV antibodies level, who received at least 24 natalizumab doses and previously treated with immunosuppressive drugs. If patients with high anti-JCV antibodies levels decide to continue natalizumab, they should undergo a higher frequency of brain MRI monitoring, in order to detect early a PML development [76]. Considering the possible fluctuation of anti-JCV antibodies level, it is strongly recommended that a serial anti-JCV antibody index testing is incorporated into standard safety monitoring protocols during natalizumab treatment in patients with a low or intermediate index value [76]. Highly active RRMS patients who received natalizumab, often show a return to pretreatment levels in term of both clinical and radiological disease activity, with a peak within 4–7 months after natalizumab withdrawal [46]. A single study [54] tried to evaluate the best way to interrupt natalizumab, showing a higher rate of relapses in the group with sudden natalizumab discontinuation compared to the one who tapered it down slowly. These interesting data suggests a way to reduce the return to high activity after natalizumab withdrawal, but more data on larger cohorts are needed.

The current options are a prolonged natalizumab “drug holiday” or a switch to a first or second line therapy for MS. Based on the review of the above-discussed data [43,46,48,49,51,52,53,54,55], “drug holiday” cannot be recommended, since this choice could lead the patient to experience severe disease reactivation and even a rebound condition, dangerous in terms of disability accumulation and also life-threatening.

The option to switch to a first line therapy is still highly controversial; some studies [49,51,77] suggest that switching to a first line treatment, like IFN or GA, significantly increases the risk of an increased MS activity. Clerico and colleagues propose that there is no difference between switching to a first line therapy or remain in a “drug holiday” status. Only few data are available regarding the most recent first line therapies, like dymethil-fumarate and teriflunomide. 

Regarding the second line therapies, fingolimod seems to be a good option in terms of efficacy when compared to IFN or GA, as reported by Iaffaldano and colleagues [56]. Kappos and colleagues [44] suggest reducing the wash out period from natalizumab to fingolimod, in order to reduce the occurrence of disease activity. However, it is important to remember that some cases of PML are reported in MS patients with fingolimod, even not previously treated with natalizumab [78]. In this context, it would be important to evaluate the opportunity to consider fingolimod as an “escape therapy” from natalizumab in JCV positive patients. A Swedish study [60] suggests an effective role of rituximab after natalizumab, but this treatment is still off-label in MS. Only few data are available regarding the switch to the new monoclonal antibody alemtuzumab [61]. In this scenario of emerging first and second therapies for MS, more clinical data are urgently needed to determine the best therapeutic approach in natalizumab quitters.

Some studies [62,63] analyzed a “bridge” therapy with intravenus steroids after natalizumab; however, the results are controversial also in this case, probably because of the small sample sizes, and there is no enough evidence at the moment for the use of steroids in the prevention of relapses in natalizumab quitters, also considering the burden of side effects related to high doses of steroids [79].

An emerging issue is the management of natalizumab during pregnancy. Currently, there are few controlled data in human pregnancy and the effects of natalizumab on pregnancy still need to be determined [69,70,80], although it seems that there is not a drug related effect on pregnancy outcomes in MS patients treated with natalizumab during the early phases of pregnancy [69,70]. At the time of prescription, patients should be plenty informed of the possible consequences of the drug on pregnancy and of the need for contraception. If MS is in a stable phase, pregnancy should be planned in advance and the treatment suspension considered in a pre-conception period, according to the half-life of the drug [81].

According to other authors [71,72,73], we think it is important to analyse in larger cohorts of patients the immunological consequences of natalizumab discontinuation, in order to find out a useful biomarker for an effective therapeutic switch. 

## 4. Conclusions

Finally, given our real-life clinical experience and on the basis of our prospective study [49], we highly recommend carefully considering whether or not to continue natalizumab after the first 24 administrations in relation to the known risk factors and the MS features before natalizumab start. Thanks to the most recent and upcoming treatments options—alemtuzumab, ocrelizumab, daclizumab, and cladribine—a vertical switch should both reduce the rebound and the PML risk. For this reason, it is desirable to plan large-scale studies to evaluate the switch from natalizumab to new therapeutic options.

## References

[1] Hauser S.L., Chan J.R., Oksenberg J.R. (2013). Multiple sclerosis: Prospects and promise. Ann. Neurol..

[2] (2004). MS Prevalence: National MS Society Study for New Prevalence Estimate. http://www.nationalmssociety.org/About-the-Society/MSPrevalence.

[3] Tullman M.J. (2013). Overview of the epidemiology, diagnosis, and disease progression associated with multiple sclerosis. Am. J. Manag. Care.

[4] Ffrench-Constant C. (1994). Pathogenesis of multiple sclerosis. Lancet.

[5] Hemler M.E., Sanchez-Madrid F., Flotte T.J., Krensky A.M., Burakoff S.J., Bhan A.K., Springer T.A., Strominger J.L. (1984). Glycoproteins of 210,000 and 130,000 m.w. on activated T cells: Cell distribution and antigenic relation to components on resting cells and T cell lines. J. Immunol..

[6] Elices M.J., Osborn L., Takada Y., Crouse C., Luhowskyj S., Hemler M.E., Lobb R.R. (1990). VCAM-1 on activated endothelium inter-acts with the leukocyte integrin VLA-4 at a site distinct from the VLA-4/fibronectin binding site. Cell.

[7] Baron J.L., Madri J.A., Ruddle N.H., Hashim G., Janeway C.A. (1993). Surface expression of alpha 4 integrin by CD4 T cells is required for their entry into brain parenchyma. J. Exp. Med..

[8] Lobb R., Hemler M. (1994). The pathophiologic role of alpha 4 integrins in vivo. J. Clin. Investig..

[9] Bayless K.J., Meininger G.A., Scholtz J.M., Davis G.E. (1998). Osteopontin is a ligand for the α4β1 integrin. J. Cell Sci..

[10] Davis L.S., Oppenheimer-Marks N., Bednarczyk J.L., McIntyre B.W., Lipsky P.E. (1990). Fibronectin promotes proliferation of naive and memory T cells by signaling through both the VLA-4 and VLA-5 integrin molecules. J. Immunol..

[11] Yednock T.A., Cannon C., Fritz L.C., Sanchez-Madrid F., Steinmant L., Karin N. (1992). Prevention of experimental autoimmune encephalomyelitis by antibodies against α4β1 integrin. Nature.

[12] Polman C.H., O’Connor P.W., Havrdova E., Hutchinson M., Kappos L., Miller D.H., Phillips J.T., Lublin F.D., Giovannoni G., Wajgt A. (2006). A randomized, placebo-controlled trial of natalizumab for relapsing multiple sclerosis. N. Engl. J. Med..

[13] Rudick R.A., Stuart W.H., Calabresi P.A., Confavreux C., Galetta S.L., Radue E.-W., Lublin F.D., Weinstock-Guttman B., Wynn D.R., Lynn F. (2006). Natalizumab plus Interferon Beta-1a for Relapsing Multiple Sclerosis. N. Engl. J. Med..

[14] Langer-Gould A., Atlas S.W., Green A.J., Bollen A.W., Pelletier D. (2005). Progressive multifocal leukoencephalopathy in a patient treated with natalizumab. N. Engl. J. Med..

[15] Kleinschmidt-DeMasters B., Tyler K.L., Bkk-d N. (2005). Progressive Multifocal Leukoencephalopathy Complicating Treatment with Natalizumab and Interferon Beta-1a for Multiple Sclerosis. N. Engl. J. Med..

[16] Van Assche G., Van Ranst M., Sciot R., Dubois B., Vermeire S., Noman M., Verbeeck J., Geboes K., Robberecht W., Rutgeerts P. (2005). Progressive multifocal leukoencephalopathy after natalizumab therapy for Crohn’s disease. N. Engl. J. Med..

[17] Avasarala J. (2016). The TOUCH program and natalizumab: Fundamental flaw in patient protection. F1000Research.

[18] Number of PML Diseases under Tysabri. https://chefarztfrau.de/?page_id=716.

[19] Baldwin K.J., Hogg J.P. (2013). Progressive multifocal leukoencephalopathy in patients with multiple sclerosis. Curr. Opin. Neurol..

[20] Sørensen P.S., Bertolotto A., Edan G., Giovannoni G., Gold R., Havrdova E., Kappos L., Kieseier B.C., Montalban X., Olsson T. (2012). Risk stratification for progressive multifocal leukoencephalopathy in patients treated with natalizumab. Mult. Scler. J..

[21] Ferenczy M.W., Marshall L.J., Nelson C.D., Atwood W.J., Nath A., Khalili K., Major E.O. (2012). Molecular biology, epidemiology, and pathogenesis of progressive multifocal leukoencephalopathy, the JC virus-induced demyelinating disease of the human brain. Clin. Microbiol. Rev..

[22] Tan C.S., Koralnik I.J. (2010). Progressive multifocal leukoencephalopathy and other disorders caused by JC virus: Clinical features and pathogenesis. Lancet Neurol..

[23] Yaldizli Ö., Putzki N. (2009). Review: Natalizumab in the treatment of multiple sclerosis. Ther. Adv. Neurol. Disord..

[24] Sabath B.F., Major E.O. (2002). Traffic of JC virus from sites of initial infection to the brain: The path to progressive multifocal leukoencephalopathy. J. Infect. Dis..

[25] Chesters P.M., Heritage J., McCance D.J. (1983). Persistence of DNA sequences of BK virus and JC virus in normal human tissues and in diseased tissues. J. Infect. Dis..

[26] Monaco M.C., Atwood W.J., Gravell M., Tornatore C.S., Major E.O. (1996). JC virus infection of hematopoietic progenitor cells, primary B lymphocytes, and tonsillar stromal cells: Implications for viral latency. J. Virol..

[27] Berger J.R., Major E.O. (1999). Progressive multifocal leukoencephalopathy. Semin. Neurol..

[28] Ledoux S., Libman I., Robert F., Just N. (1989). Progressive multifocal leukoencephalopathy with gray matter involvement. Can. J. Neurol. Sci..

[29] Richardson E.P., Webster H.D. (1983). Progressive multifocal leukoencephalopathy: Its pathological features. Prog. Clin. Biol. Res..

[30] Du Pasquier R.A., Corey S., Margolin D.H., Williams K., Pfister L.A., De Girolami U., Mac Key J.J., Wüthrich C., Joseph J.T., Koralnik I.J. (2003). Productive infection of cerebellar granule cell neurons by JC virus in an HIV^+^ individual. Neurology.

[31] Wüthrich C., Dang X., Westmoreland S., McKay J., Maheshwari A., Anderson M.P., Ropper A.H., Viscidi R.P., Koralnik I.J. (2009). Fulminant JC virus encephalopathy with productive infection of cortical pyramidal neurons. Ann. Neurol..

[32] Berger J.R., Aksamit A.J., Clifford D.B., Davis L., Koralnik I.J., Sejvar J.J., Bartt R., Major E.O., Nath A. (2013). PML diagnostic criteria: Consensus statement from the AAN neuroinfectious disease section. Neurology.

[33] Mentzer D., Prestel J., Adams O., Gold R., Hartung H.-P., Hengel H., Kieseier B.C., Ludwig W.D., Keller-Stanislawski B. (2012). Case definition for progressive multifocal leukoencephalopathy following treatment with monoclonal antibodies. J. Neurol. Neurosurg. Psychiatry.

[34] Pavlovic D., Patera A.C., Nyberg F., Gerber M., Liu M. (2015). Progressive Multifocal Leukeoncephalopathy Consortium. Progressive multifocal leukoencephalopathy: Current treatment options and future perspectives. Ther. Adv. Neurol. Disord..

[35] Khatri B.O., Man S., Giovannoni G., Koo A.P., Lee J.C., Tucky B., Lynn F., Jurgensen S., Woodworth J., Goelz S. (2009). Effect of plasma exchange in accelerating natalizumab clearance and restoring leukocyte function. Neurology.

[36] Subramanyam M., Plavina T., Khatri B.O., Fox R.J., Goelz S.E. (2013). The effect of plasma exchange on serum anti-JC virus antibodies. Mult. Scler. J..

[37] Plavina T., Subramanyam M., Bloomgren G., Richman S., Pace A., Lee S., Schlain B., Campagnolo D., Belachew S., Ticho B. (2014). Anti-JC virus antibody levels in serum or plasma further define risk of natalizumab-associated progressive multifocal leukoencephalopathy. Ann. Neurol..

[38] EMA Confirms Recommendations to Minimise Risk of Brain Infection PML with Tysabri. http://www.ema.europa.eu/docs/en_GB/document_library/Press_release/2016/02/WC500202389.pdf.

[39] European Medicines Agency European Medicines Agency Recommends Additional Measures to Better Manage Risk of Progressive Multifocal Leukoencephalopathy (PML) with Tysabri. http://www.ema.europa.eu/ema/index.jsp?curl=pages/news_and_events/news/2010/01/news_detail_000987.jsp&mid=WC0b01ac058004d5c1.

[40] O’Connor P.W. (2011). ‘Disease activity return during natalizumab treatment interruption in patients with multiple sclerosis’: Author response. Neurology.

[41] Berger J.R., Centonze D., Comi G., Confavreux C., Cutter G., Giovannoni G., Gold R., Hartung H.-P., Lublin F., Miravalle A. (2010). Considerations on discontinuing natalizumab for the treatment of multiple sclerosis. Ann. Neurol..

[42] Sangalli F., Moiola L., Ferrè L., Radaelli M., Barcella V., Rodegher M., Colombo B., Martinelli Boneschi F., Martinelli V., Comi G. (2014). Long-term management of natalizumab discontinuation in a large monocentric cohort of multiple sclerosis patients. Mult. Scler. Relat. Disord..

[43] Killestein J., Vennegoor A., Strijbis E.M., Seewann A., Van Oosten B.W., Uitdehaag B.M.J., Polman C.H. (2010). Natalizumab drug holiday in multiple sclerosis: Poorly tolerated. Ann. Neurol..

[44] Kappos L., Radue E.W., Comi G., Montalban X., Butzkueven H., Wiendl H., Giovannoni G., Hartung H.P., Derfuss T., Naegelin Y. (2015). Switching from natalizumab to fingolimod: A randomized, placebo-controlled study in RRMS. Neurology.

[45] Stüve O., Marra C.M., Jerome K.R., Cook L., Cravens P.D., Cepok S., Frohman E.M., Phillips J.T., Arendt G., Hemmer B. (2006). Immune surveillance in multiple sclerosis patients treated with natalizumab. Ann. Neurol..

[46] O’Connor P.W., Goodman A., Kappos L., Lublin F.D., Miller D.H., Polman C., Rudick R.A., Aschenbach W., Lucas N. (2011). Disease activity return during natalizumab treatment interruption in patients with multiple sclerosis. Neurology.

[47] Goodman A.D., Rossman H., Bar-Or A., Miller A., Miller D.H., Schmierer K., Lublin F., Khan O., Bormann N.M., Yang M. (2009). GLANCE: Results of a phase 2, randomized, double-blind, placebo-controlled study. Neurology.

[48] Fox R.J., Cree B.A.C., De Sèze J., Gold R., Hartung H.P., Jeffery D., Kappos L., Kaufman M., Montalban X., Weinstock-Guttman B. (2014). MS disease activity in RESTORE: A randomized 24-week natalizumab treatment interruption study. Neurology.

[49] Clerico M., Schiavetti I., De Mercanti S.F., Piazza F., Gned D., Brescia Morra V., Lanzillo R., Ghezzi A., Bianchi A., Salemi G. (2014). Treatment of Relapsing-Remitting Multiple Sclerosis After 24 Doses of Natalizumab: Evidence From an Italian Spontaneous, Prospective, and Observational Study (the TY-STOP Study). JAMA Neurol..

[50] Sorensen P.S., Koch-Henriksen N., Petersen T., Ravnborg M., Oturai A., Sellebjerg F. (2014). Recurrence or rebound of clinical relapses after discontinuation of natalizumab therapy in highly active MS patients. J. Neurol..

[51] Lo Re M., Capobianco M., Ragonese P., Realmuto S., Malucchi S., Berchialla P., Salemi G., Bertolotto A. (2015). Natalizumab Discontinuation and Treatment Strategies in Patients with Multiple Sclerosis (MS): A Retrospective Study from Two Italian MS Centers. Neurol. Ther..

[52] Papeix C., Vukusic S., Casey R., Debard N., Stankoff B., Mrejen S., Uhry Z., van Ganse E., Castot A., Clanet M. (2016). Risk of relapse after natalizumab withdrawal: Results from the French TYSEDMUS cohort. Neurol. Neuroimmunol. Neuroinflamm..

[53] Iaffaldano P., Viterbo R.G., Trojano M. (2016). Natalizumab discontinuation is associated with a rebound of cognitive impairment in multiple sclerosis patients. J. Neurol..

[54] Weinstock-Guttman B., Hagemeier J., Kavak K.S., Saini V., Patrick K., Ramasamy D.P., Nadeem M., Carl E., Hojnacki D., Zivadinov R. (2016). Randomised natalizumab discontinuation study: Taper protocol may prevent disease reactivation. J. Neurol. Neurosurg. Psychiatry.

[55] Vellinga M.M., Castelijns J.A., Barkhof F., Uitdehaag B.M.J., Polman C.H. (2008). Postwithdrawal rebound increase in T2 lesional activity in natalizumab-treated MS patients. Neurology.

[56] Iaffaldano P., Lucisano G., Pozzilli C., Brescia Morra V., Ghezzi A., Millefiorini E., Patti F., Lugaresi A., Zimatore G.B., Marrosu M.G. (2015). Fingolimod versus interferon beta/glatiramer acetate after natalizumab suspension in multiple sclerosis. Brain.

[57] Villaverde-González R., Gracia Gil J., Pérez Sempere A., Millán Pascual J., Marín Marín J., Carcelén Gadea M., Gabaldón Torres L., Moreno Escribano A., Candeliere Merlicco A. (2017). Observational Study of Switching from Natalizumab to Immunomodulatory Drugs. Eur. Neurol..

[58] Patti F., Leone C., Zappia M. (2015). Clinical and radiologic rebound after discontinuation of natalizumab therapy in a highly active multiple sclerosis patient was not halted by dimethyl-fumarate: A case report. BMC Neurol..

[59] Ferrè L., Moiola L., Sangalli F., Radaelli M., Barcella V., Comi G., Martinelli V. (2015). Recurrence of disease activity after repeated Natalizumab withdrawals. Neurol. Sci..

[60] Alping P., Frisell T., Novakova L., Islam-Jakobsson P., Salzer J., Björck A., Axelsson M., Malmeström C., Fink K., Lycke J. (2016). Rituximab versus fingolimod after natalizumab in multiple sclerosis patients. Ann. Neurol..

[61] Malucchi S., Capobianco M., Lo Re M., Malentacchi M., di Sapio A., Matta M., Sperli F., Bertolotto A. (2016). High-Risk PML Patients Switching from Natalizumab to Alemtuzumab: An Observational Study. Neurol. Ther..

[62] Borriello G., Prosperini L., Mancinelli C., Giannì C., Fubelli F., Pozzilli C. (2012). Pulse monthly steroids during an elective interruption of natalizumab: A post-marketing study. Eur. J. Neurol..

[63] Evangelopoulos M.E., Koutoulidis V., Andreadou E., Evangelopoulos D.S., Kilidireas C. (2016). Pulsed corticosteroid treatment in MS patients stabilizes disease activity following natalizumab withdrawal prior to switching to fingolimod. Int. J. Neurosci..

[64] Rossi S., Motta C., Studer V., Boffa L., De Chiara V., Castelli M., Barbieri F., Buttari F., Monteleone F., Germani G. (2014). Treatment options to reduce disease activity after natalizumab: Paradoxical effects of corticosteroids. CNS Neurosci. Ther..

[65] Buraga I., Popovici R.E. (2014). Multiple sclerosis and pregnancy: Current considerations. Sci. World J..

[66] Fagius J., Burman J. (2014). Normal outcome of pregnancy with ongoing treatment with natalizumab. Acta Neurol. Scand..

[67] Schneider H., Weber C.E., Hellwig K., Schroten H., Tenenbaum T. (2013). Natalizumab treatment during pregnancy-effects on the neonatal immune system. Acta Neurol. Scand..

[68] Haghikia A., Langer-Gould A., Rellensmann G., Schneider H., Tenenbaum T., Elias-Hamp B., Menck S., Zimmermann J., Herbstritt S., Marziniak M. (2014). Natalizumab use during the third trimester of pregnancy. JAMA Neurol..

[69] Ebrahimi N., Herbstritt S., Gold R., Amezcua L., Koren G., Hellwig K. (2015). Pregnancy and fetal outcomes following natalizumab exposure in pregnancy. A prospective, controlled observational study. Mult. Scler. J..

[70] Friend S., Richman S., Bloomgren G., Cristiano L.M., Wenten M., Alcalde-Cabero E., Almazán-Isla J., García-Merino A., Sá J., Pedro-Cuesta J. (2016). Evaluation of pregnancy outcomes from the Tysabri^®^ (natalizumab) pregnancy exposure registry: A global, observational, follow-up study. BMC Neurol..

[71] Larochelle C., Metz I., Lecuyer M.-A., Terouz S., Roger M., Arbour N., Bruck W., Prat A. (2017). Immunological and pathological characterization of fatal rebound MS activity following natalizumab withdrawal. Mult. Scler. J..

[72] Haas J., Schneider K., Schwarz A., Korporal-Kuhnke M., Faller S., von Glehn F., Jarius S., Wildemann B. (2017). Th17 cells: A prognostic marker for MS rebound after natalizumab cessation?. Mult. Scler. J..

[73] Harrer A., Pilz G., Oppermann K., Sageder M., Afazel S., Haschke-Becher E., Rispens T., de Vries A., McCoy M., Stevanovic V. (2017). From natalizumab to fingolimod in eight weeks-Immunological, clinical, and radiological data in quest of the optimal switch. Clin. Immunol..

[74] O’Connor P., Goodman A., Kappos L., Lublin F., Polman C., Rudick R.A., Hauswirth K., Cristiano L. M., Forrestal F., Duda P. (2014). Long-term safety and effectiveness of natalizumab redosing and treatment in the STRATA MS study. Neurology.

[75] Butzkueven H., Kappos L., Pellegrini F., Trojano M., Wiendl H., Patel R.N., Zhang A., Hotermans C., Belachew S., Butzkueven H. (2014). Efficacy and safety of natalizumab in multiple sclerosis: Interim observational programme results. J. Neurol. Neurosurg. Psychiatry.

[76] McGuigan C., Craner M., Guadagno J., Kapoor R., Mazibrada G., Molyneux P., Nicholas R., Palace J., Pearson O.R., Rog D. (2016). Stratification and monitoring of natalizumab-associated progressive multifocal leukoencephalopathy risk: Recommendations from an expert group. J. Neurol. Neurosurg. Psychiatry.

[77] Sangalli F., Moiola L., Bucello S., Annovazzi P., Rizzo A., Radaelli M., Vitello G., Grimaldi L.M.E., Ghezzi A., Martinelli V. (2011). Efficacy and tolerability of natalizumab in relapsing-remitting multiple sclerosis patients: A post-marketing observational study. J. Neurol. Sci..

[78] Misbah S.A. (2017). Progressive multifocal leukoencephalopathy-driven from rarity to clinical mainstream by iatrogenic immunodeficiency. Clin. Exp. Immunol..

[79] Oliveira A.T., Lopes S., Cipriano M.A., Sofia C. (2015). Induced liver injury after high-dose methylprednisolone in a patient with multiple sclerosis. BMJ Case Rep..

[80] Lu E., Wang B.W., Guimond C., Synnes A., Sadovnick D., Tremlett H. (2012). Disease-Modifying drugs for multiple sclerosis in pregnancy; A systematic review. Neurology.

[81] Leroy C., Rigot J.-M., Leroy M., Decanter C., Le Mapihan K., Parent A.-S., Le Guillou A.-C., Yakoub-Agha I., Dharancy S., Noel C. (2015). Immunosuppressive drugs and fertility. Orphanet J. Rare Dis..

